# Impact of a 40-Gene Targeted Panel Test on Physician Decision Making for Patients With Acute Myeloid Leukemia

**DOI:** 10.1200/PO.20.00182

**Published:** 2021-01-14

**Authors:** Erica K. Barnell, Kenneth F. Newcomer, Zachary L. Skidmore, Kilannin Krysiak, Sydney R. Anderson, Lukas D. Wartman, Stephen T. Oh, John S. Welch, Keith E. Stockerl-Goldstein, Ravi Vij, Amanda F. Cashen, Iskra Pusic, Peter Westervelt, Camille N. Abboud, Armin Ghobadi, Geoffrey L. Uy, Mark A. Schroeder, John F. Dipersio, Mary C. Politi, David H. Spencer, Eric J. Duncavage, Timothy J. Ley, Malachi Griffith, Meagan A. Jacoby, Obi L. Griffith

**Affiliations:** ^1^ McDonnell Genome Institute, Washington University School of Medicine, St Louis, MO; ^2^ Department of Surgery, Washington University School of Medicine, St Louis, MO; ^3^ Department of Medicine, Division of Oncology, Washington University School of Medicine, St Louis, MO; ^4^ Siteman Cancer Center, Washington University School of Medicine, St Louis, MO; ^5^ Department of Medicine, Division of Hematology, Washington University School of Medicine, St Louis, MO; ^6^ Department of Surgery, Division of Public Health Sciences, Washington University School of Medicine, St Louis, MO; ^7^ Department of Pathology and Immunology, Washington University School of Medicine, St Louis, MO; ^8^ Department of Genetics, Washington University School of Medicine, St Louis, MO

## Abstract

**PURPOSE:**

Physicians treating hematologic malignancies increasingly order targeted sequencing panels to interrogate recurrently mutated genes. The precise impact of these panels on clinical decision making is not well understood.

**METHODS:**

Here, we report our institutional experience with a targeted 40-gene panel (MyeloSeq) that is used to generate a report for both genetic variants and variant allele frequencies for the treating physician (the limit of mutation detection is approximately one AML cell in 50).

**RESULTS:**

In total, 346 sequencing reports were generated for 325 patients with suspected hematologic malignancies over an 8-month period (August 2018 to April 2019). To determine the influence of genomic data on clinical care for patients with acute myeloid leukemia (AML), we analyzed 122 consecutive reports from 109 patients diagnosed with AML and surveyed the treating physicians with a standardized questionnaire. The panel was ordered most commonly at diagnosis (61.5%), but was also used to assess response to therapy (22.9%) and to detect suspected relapse (15.6%). The panel was ordered at multiple timepoints during the disease course for 11% of patients. Physicians self-reported that 50 of 114 sequencing reports (44%) influenced clinical care decisions in 44 individual patients. Influences were often nuanced and extended beyond identifying actionable genetic variants with US Food and Drug Administration–approved drugs.

**CONCLUSION:**

This study provides insights into how physicians are currently using multigene panels capable of detecting relatively rare AML cells. The most influential way to integrate these tools into clinical practice will be to perform prospective clinical trials that assess patient outcomes in response to genomically driven interventions.

## INTRODUCTION

Integrating individualized genomic information into care to improve patient outcomes is an area of interest within oncology.^1^ Methods for obtaining genomic information include internal testing, which typically occurs at larger academic institutions, or outsourced assessment through centralized commercial laboratories. Genomic data can be obtained from single-gene testing, targeted sequencing panels, or comprehensive—whole genome or whole exome—sequencing approaches.

**CONTEXT**

**Key Objective**
Whereas physicians have increased the use of targeted sequencing panels to inform treatment decisions for patients with hematologic malignancies, the impact and utility of sequencing data on clinical decision making is not well understood. Through standardized surveys, this study captured the nuanced impact of sequencing data on clinical decision making for patients with acute myeloid leukemia.
**Knowledge Generated**
Physicians self-reported that 50 of 114 sequencing reports (44%) influenced clinical care decisions with 56 total influences reported, 38 of which were related to therapeutic options, 10 related to risk stratification in first clinical remission, and eight involving measuring persistent molecular disease. In 11% of all patients, the panel was ordered at multiple timepoints during the disease course for persistent molecular disease monitoring.
**Relevance**
This study demonstrates that physicians are integrating sequencing data obtained at various points in the acute myeloid leukemia disease course into clinical decision making, mostly to determine therapy choices, stratify relapse risk, and measure persistent disease.

All sequencing data used for cancer assessment requires variant identification and annotation. Some single-gene tests and two multitarget panels are currently US Food and Drug Administration (FDA) approved and have associated companion diagnostics that designate a specific cancer subtype and indication.^2^ However, the vast majority of variant identification and annotation is performed by laboratory developed tests (LDTs),^3^ for which results are provided as a clinical report. These reports typically require a certain level of genomic literacy to understand and implement.^4,5^ Lack of report standardization, inadequate genomic training,^6,7^ limited infrastructure to support oncologists,^1,8^ and limited resources—for example, low reimbursement rates and lack of access to clinical trials—are thought to hinder the impact of sequencing data on clinical care.^9,10^ The result is a discrepancy between the identification of clinically actionable variants^11^ and implementation of change in treatment decisions.^1,12^

Acute myeloid leukemia (AML) sequencing data are particularly complex because of the genetic and clonal heterogeneity of AML cases. More than 250 recurrently mutated genes and specific hotspot variants have been described in AML,^13^ and prognostic significance may depend on cooperating covariants in multiple other genes.^14^ For example, European LeukemiaNet/National Comprehensive Cancer Network guidelines specifically mention six genes/mutations in their risk stratification guidelines, all of which must be evaluated in parallel for risk stratification.^15,16^ In addition, whereas pathogenic variants within three genes—*FLT3*, *IDH2*, and *IDH1*—have been used as predictive markers for FDA-approved targeted therapeutics,^17-19^ ongoing clinical trials have demonstrated predictive utility for other pathogenic variants that do not yet have approved companion diagnostics.^20^

Next-generation sequencing (NGS) data can provide information beyond therapeutic sensitivity/resistance that is not currently captured by FDA-approved diagnostics. Specifically, quantification of the variant allele frequency (VAF) of tumor-associated variants has been used to detect persistent molecular disease (PMD) after therapies are administered, and predict both relapse risk^21^ and survival outcomes.^22-24^ Although multipanel LDT diagnostics are being used on patients with AML to supplement treatment decisions, the utility of these reports is not well described.

This study sought to better understand the extent to which genomic reports influence clinical decision making for patients with AML. This paper describes physician experience with a targeted gene panel (MyeloSeq) that interrogates 40 recurrently mutated genes or gene hotspots and returns gene variant and VAF data to the treating physician. We determined usage patterns over an 8-month period and surveyed treating physicians regarding how they used the panel results in clinical care.

## METHODS

### Study Design and Patient Eligibility

This study was conducted at Washington University School of Medicine (Human Research Protection Office #201801112). We aimed to study approximately 100 patients with AML in less than 1 year after the initiation of the study. All consecutive reports ordered by a physician at any point in a patient’s treatment between August 17, 2018, and April 9, 2019, were analyzed. The panel was a targeted sequencing assay that evaluated 40 genes and gene hotspots that are recurrently mutated in myeloid malignancies (Data Supplement).^25^ For each patient who was found to have a definitive diagnosis of AML, a survey was sent to the treating physician. Patients with acute promyelocytic leukemia (M3 AML subtype) were excluded.^26^ The treating physician was asked to complete a nine-question survey to determine how the physician used the panel to inform treatment decisions (Data Supplement).

### Panel Design and Processing

The panel uses an amplicon capture-based enrichment with unique molecular identifiers for ultra-high variant sensitivity that targets an approximately 98-kb space (HaloPlex Target Enrichment System; Agilent Technologies, Santa Clara, CA). Patients with a definitive diagnosis of AML were verified by evaluating the patient’s standard diagnostic workup. This included evaluating bone marrow morphology, standard panels of flow cytometric markers, and a panel of routine cytogenetic and molecular marker studies, including fluorescence in situ hybridization probes. Target enrichment, variant calling strategies, and variant annotation methods are described in the Data Supplement.

The median number of failed genes across all reports was two (range, 0 to 24 genes). *WT1* (n = 211 cases), *CUX1* (n = 197 cases), and *CEBPA* (n = 111 cases) were genes that most frequently failed coverage requirements. Gene failure was attributable to specific gene regions that were difficult to target using existing reagents. A summary of the assay validation report is provided in the Data Supplement.

### Variant Annotation and Physician Survey

Reports were generated using annotated variant call format files, binary alignment map files, preliminary clinical annotation, and quality metrics. Reports were reviewed by faculty members from the Washington University Department of Pathology and Immunology and signed out within the secure network. The final report was integrated into the patient’s electronic medical record for review by the treating physician. Physicians were surveyed after reports were issued using the standardized electronic questionnaire (Data Supplement). If physicians did not respond to the initial contact, reminders were provided to increase the response rate.

### Ethics, Consent, Permissions, and Consent to Publish

This study was conducted at Washington University School of Medicine and was approved by the Washington University Human Research Protection Office (#201801112). Consent information sheets were sent to all individuals who participated in the study. This information sheet included information on consent to publish. All reasonable steps were taken to ensure confidentiality of participant information.

### Code and Data Accessibility

Figures and data analysis were performed using python (v 3.6.1), R (v 3.5.2), and Anaconda (v 4.3.0). Figures were generated using seaborn (v 0.8.1), SankeyMATIC (v BETA), and GenVisR (v 1.15.3).^27^ Sequencing panel data and survey data are made available in the Data Supplement. Raw sequencing data from reports were not made available for patient privacy reasons.

## RESULTS

### Identification of Variants in Myeloid Malignancies

**FIG 1.**
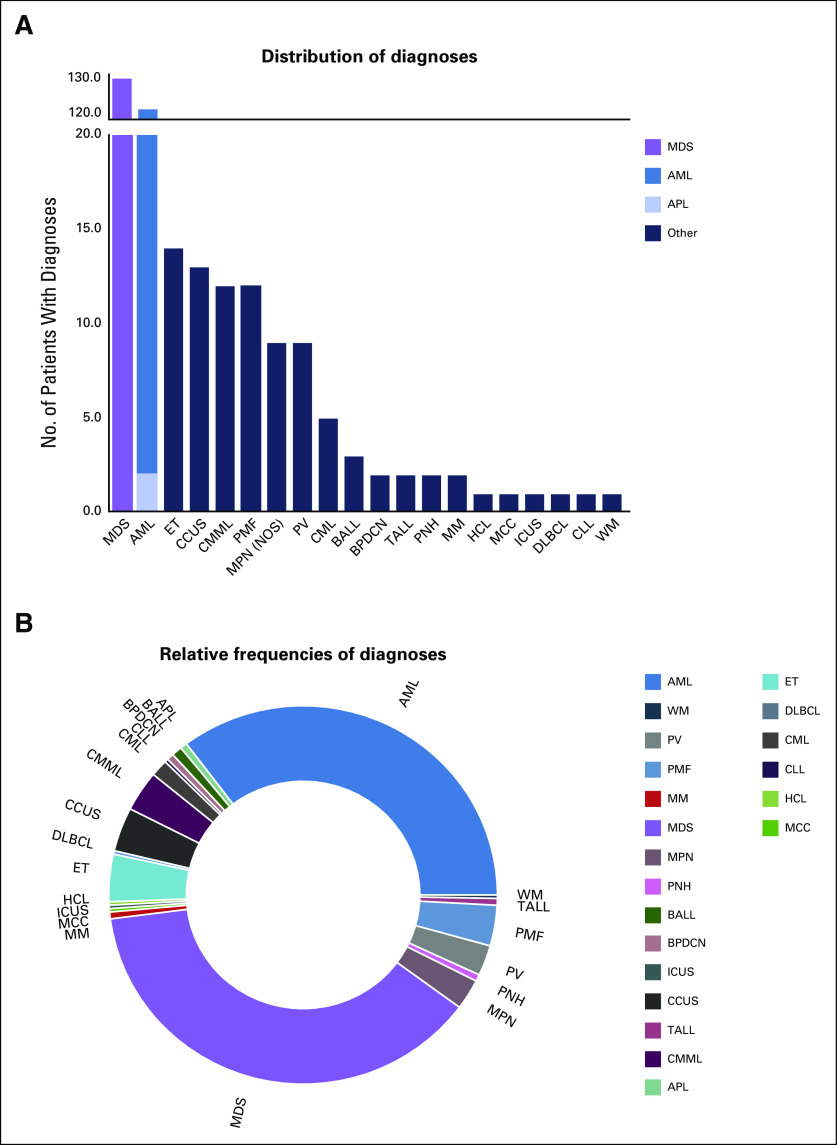
Distribution of patient diagnoses. (A) Distribution and (B) relative frequency of hematologicb disorders for the 346 samples evaluated with the sequencing panel. Diagnosis is based on the clinical history ascertained by pathologists signing out the report. Acute myeloid leukemia (AML) with acute promyelocytic leukemia (APL) subtype—designated by a light blue bar under the AML distribution bar chart—were not eligible for analysis with the sequencing panel. BALL, B-cell acute lymphoblastic leukemia; BPDCN, blastic plasmacytoid dendritic cell neoplasm; CCUS, clonal cytopenia of undetermined significance; CML, chronic myeloid leukemia; CMML, chronic myelomonocytic leukemia; CLL, chronic lymphoblastic leukemia; DLBCL, diffuse large B-cell lymphoma; ET, essential thrombocytopenia; HCL, hairy cell leukemia; ICUS, idiopathic cytopenias of undetermined significance; MCC, Merkel cell carcinoma; MDS, myelodysplastic syndrome; MM, multiple myeloma; MPN, myeloproliferative neoplasm, not otherwise specified; PMF, primary myelofibrosis; PNH, paroxysmal nocturnal hemoglobinuria; PV, polycythemia vera; TALL, T-cell acute lymphoblastic leukemia; WM, Waldenström macroglobulinemia.

Over an approximately 8-month period (August 2018 to April 2019), 346 reports from 325 unique patients were generated. The median return time was 12 days (range, 4 to 89 days). The median number of reports ordered per month was 15 (range, 11 to 30 reports). There were 824 variants observed in the 40 targeted genes across all 346 reports (Data Supplement). The distribution of patient diagnoses for which a report was generated is shown in Figures 1A and 1B. The most common diagnoses were myelodysplastic syndrome (n = 128 samples; 37%) and AML (n = 124 samples; 36%).

### Patterns of Panel Use

**FIG 2.**
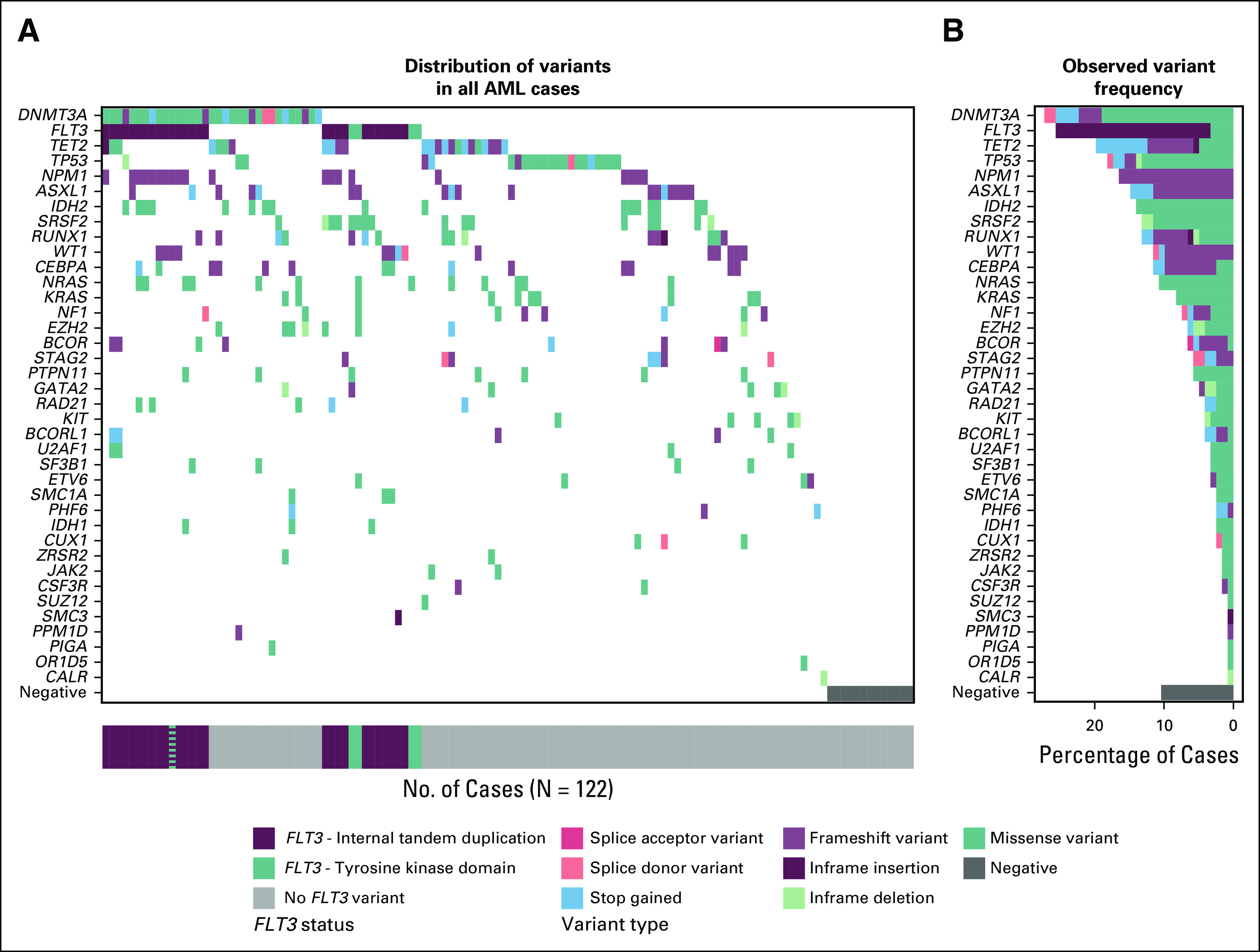
Distribution of variants in acute myeloid leukemia (AML) cases. (A) Heatmap of the distribution of variants in all AML cases reported. Each row represents a single gene and each column represents a sequencing report (N = 122). Colored squares denote that a variant was observed in the designated gene. Colors indicate the variant type. If there was more than one variant observed per gene within the same case, the most deleterious variant on the basis of the variant effect prediction^44^ was listed. The bottom bar indicates the *FLT3* status for all 122 patients. The bar color indicates the type of *FLT3* variant (internal tandem duplication *v* tyrosine kinase domain). (B) Percentage of cases with a variant in a given gene. Each row represents a single gene and the color indicates the variant type.

There were 124 reports from patients with AML. The median time from sample accession to sign out was 13.4 days (range, 4 to 36 days). The reports from patients with AML, after removing patients with APL subtype (n = 122 samples), were derived from 109 unique patients who were under the care of 14 physicians (Table 1). The distribution of variants from these patients reflected the expected AML genomic landscape^13,14^ (Fig 2). In total, 38 of 40 genes interrogated by the panel were observed in at least one AML report. Only 13 samples had no observed variants, eight of which were acquired from patients who were determined to be in clinical remission using bone marrow biopsy.

**TABLE 1.**
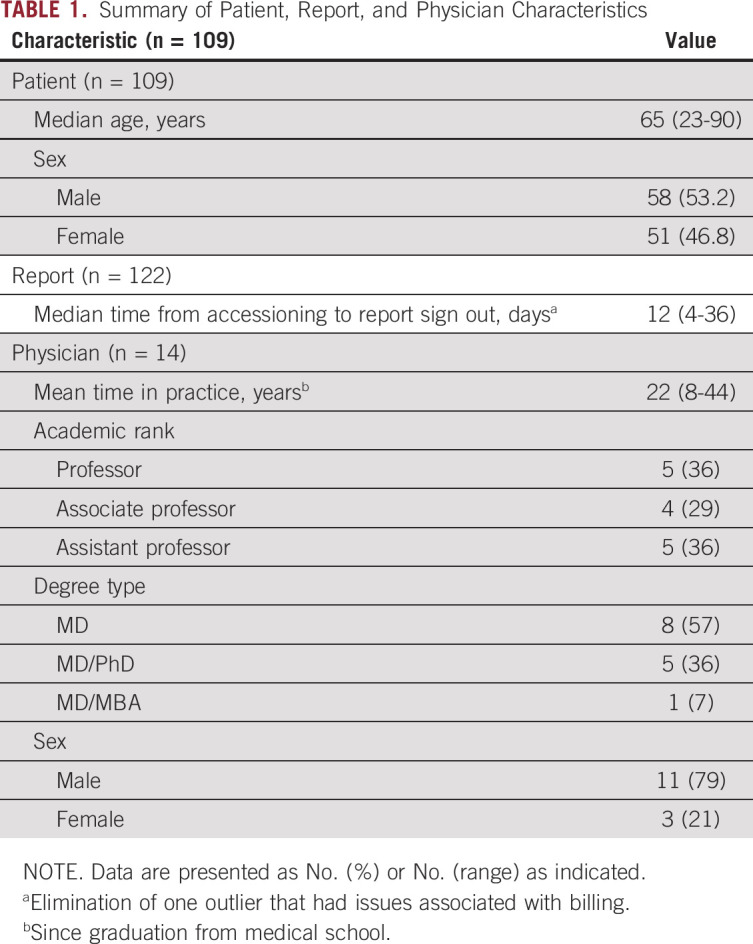
Summary of Patient, Report, and Physician Characteristics

**FIG 3.**
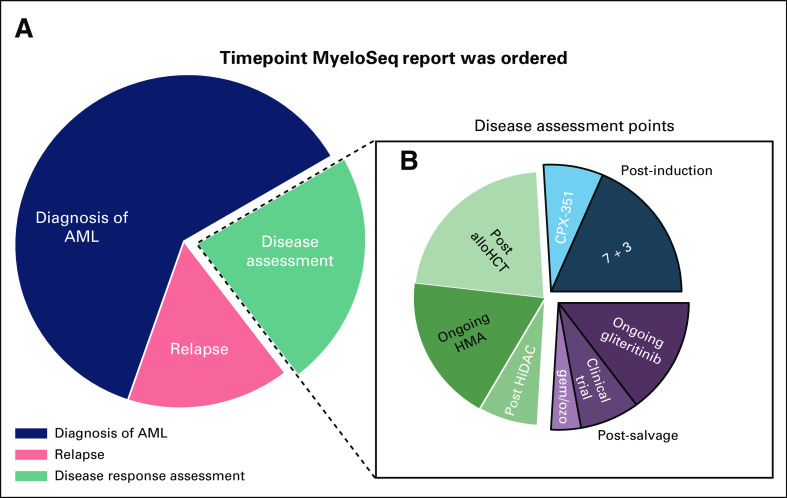
Physicians ordered sequencing panels at various times throughout the acute myeloid leukemia (AML) course. (A) Distribution of timepoints. (B) Distribution of therapeutics for which the sequencing report was ordered as part of response assessment. 7 + 3, 7 days cytarabine and 3 days anthracycline; alloHCT, allogeneic stem-cell transplantation; CPX-351, liposomal cytarabine and daunorubicin; gem/ozo, gemtuzumab ozogamicin; HiDAC, high-dose Ara-C; HMA, hypomethylating agent.

Reports were ordered at various points in the disease course. The most common timepoint was at diagnosis (n = 75 of 122 [61.5%]). The next most common timepoint was during disease assessment (n = 28 of 122; [22.9%]), followed by potential relapse (n = 19 of 122 [15.6%]; Fig 3A). For reports ordered during disease response assessments, seven were ordered post-induction, six were ordered after allogeneic hematopoietic cell transplant (alloHCT), five were ordered during ongoing treatment with a hypomethylating agent, two were ordered after consolidation with high-dose Ara-C, and seven were ordered during or after salvage therapy (Fig 3B). One case was ordered at an undefined timepoint.

### Treatment Regimens Guided by Variants

**FIG 4.**
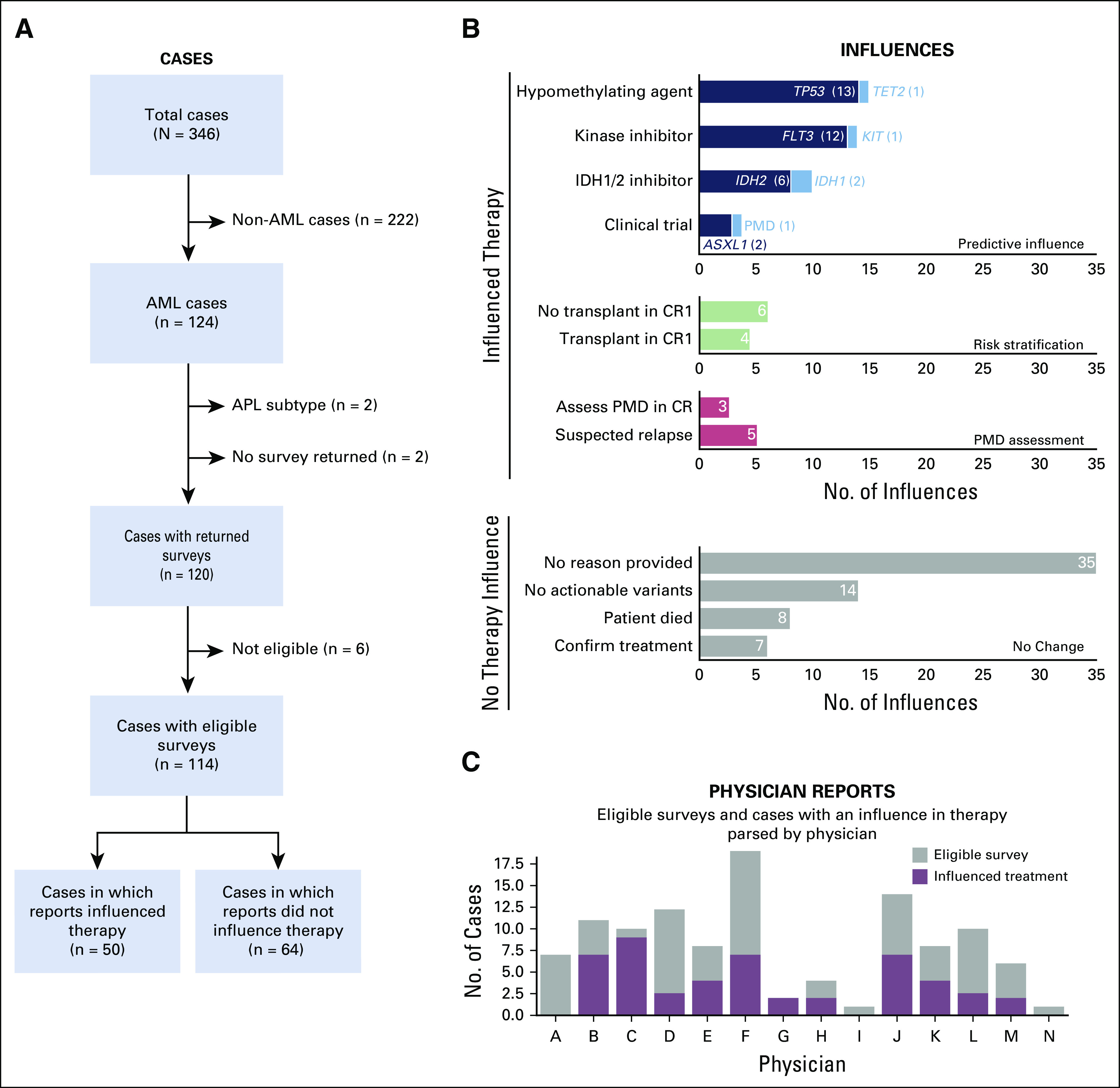
Physician-reported influences from sequencing reports issued for patients with acute myeloid leukemia (AML). (A) In total, 346 consecutive cases—that is, reports—were analyzed and 124 had a diagnosis of AML. Of these 124 cases, four were excluded (acute promyelocytic leukemia [APL] subtype [n = 2]; no survey returned [n = 2]). Six surveys were returned but ineligible for additional analysis. Physicians reported that the report influenced clinical decision making in 50 cases (44%). (B) Physician-reported influences. Variants associated with predictive influences are labeled. In 10 cases, variants observed on sequencing reports were used to stratify relapse risk for patients in first clinical remission (CR1). In eight cases, variants observed were used to assess for persistent molecular disease (PMD). In 64 cases, physicians reported that the results did not inform decision making. (C) There were 14 physicians who contributed at least one survey to this study.

Physicians provided survey responses for 120 of the 122 AML reports, a 98% response rate. Of those, six were ineligible for additional analysis—two patients were lost to follow up and four declined treatment. Of the remaining 114 reports, physicians described 56 influences from 50 reports. On the basis of these data, the sequencing report was a factor in determining therapy decisions in 50 (43.8%) of 114 cases (Fig 4A). There was no significant difference in the proportion of cases with influences among timepoints (diagnosis, relapse, and disease assessment; see Data Supplement). The patient accepted the treatment plan 93% of the time (n = 38 of 41 cases).

### Predictive Influences

In 35 of 50 reports, physicians indicated that they recommended at least one therapy on the basis of the variants identified by the sequencing panel (Fig 4B). On the basis of sequencing panel results—35 reports with 38 corresponding influences—physicians reported recommending a hypomethylating agent in 14 patients for a *TP53*^20^ or *TET2*^28,29^ variant, kinase inhibitors (midostaurin or gilteritinib) in 12 patients with a *FLT3* variant^30^ and one patient with a *KIT* variant,^31^ and *IDH1/IDH2* inhibitors were recommended for eight patients.^32^ There were three patients for whom a clinical trial was recommended on the basis of observed variants or persistent disease.

There were 31 cases with an *FLT3* variant. In 16 of 29 eligible cases (two patients refused treatment), physicians reported the following influences: 12 used the NGS panel to prescribe an *FLT3* inhibitor and four influences were independent of *FLT3* status. In three of these four cases, patients had an *FLT3* inhibitor prescribed based on a previously known result from a different genetic test. There were 13 cases in which the patient had an *FLT3* variant, but the physician reported no influence. Of these 13 cases, three patients died before treatment initiation and eight had an *FLT3* inhibitor prescribed based on a previously known result from a different genetic test (Data Supplement).

### Risk Stratification for Transplant in First Clinical Remission

Physicians identified 10 reports that influenced patient risk assessment and alloHCT recommendation (Fig 4B). Specifically, alloHCT was not recommended in first complete remission in six patients, based on the presence of bialleleic *CEBPA* variants (n = 2), *KIT* variant status (n = 1), lack of high-risk variants (n = 1), or differential clearance of co-occurring variants after induction therapy (n = 1). For the latter patient, three variants were detected at diagnosis (*DNMT3A* [VAF = 42%], *NPM1* [VAF = 38%], and *RAD21* [VAF = 43%]). However, postinduction, the sequencing panel showed persistence of the *DNMT3A* R882H variant with a high VAF (43%). For this patient, postconsolidation PMD testing by flow cytometry^33^ was negative. The physician reported that the clearance of all previous variants and the patient’s cytogenetic abnormality, with the exception of the *DNMT3A* R882H variant, suggested that the variant is evidence of an age-related clonal hematopoiesis with an increased risk of relapse over time.

In contrast, there were four patients for whom physicians reported recommending alloHCT in first complete remission. These recommendations were based on *TP53* variant status (n = 2), *DNMT3A* variant status (n = 1), and co-occurrence of multiple variants (*DNMT3A*, *NPM1*, and *FLT3* internal tandem duplication; n = 1).^14^

### Assessment for PMD

**FIG 5.**
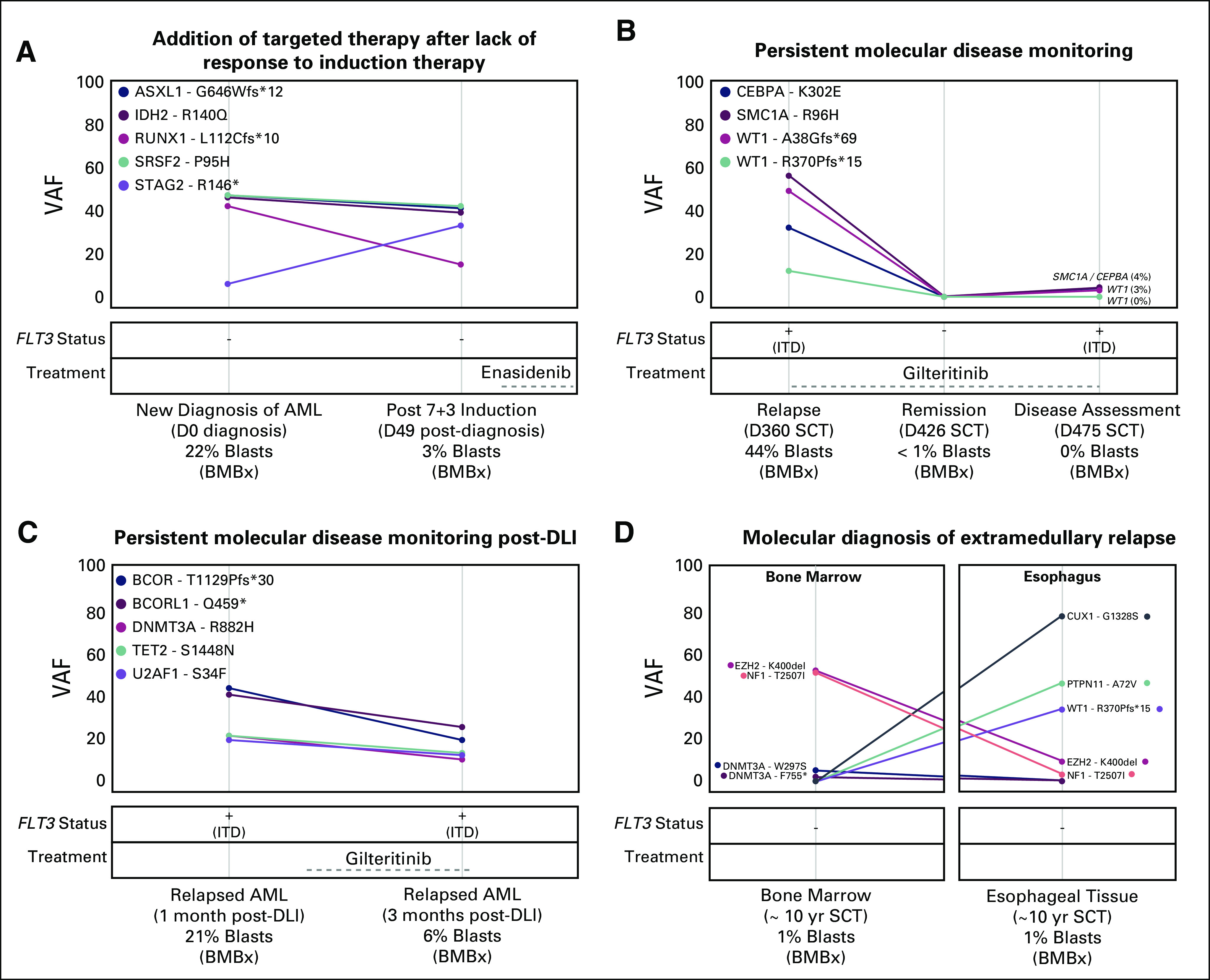
Incorporation of panel testing in disease monitoring for patients with acute myeloid leukemia (AML). Each panel represents a single patient where multiple sequencing reports were obtained. The plot indicates any variants observed with associated variant allele frequencies (VAFs) at the timepoints labeled on the *x*-axis. These plots also show *FLT3* variant status (presence tyrosine kinase domain or internal tandem duplication [ITD]) and approximate duration of treatment. (A) Sequencing panels revealed persistent molecular disease after induction. Based on the persistent *IDH2* variant, the physician initiated targeted therapy with enasidenib. (B) Physician reported that the observation of persistent molecular disease at 475 days (D) post–stem-cell transplantation (SCT) indicated the need for a second allogeneic stem-cell transplantation despite clinical remission (0% blasts on bone marrow biopsy [BMBx]). (C) Sequencing panels were used to track VAFs to evaluate the efficacy of a donor lymphocyte infusion (DLI) and gilteritinib over time. (D) Extramedullary relapse was confirmed by comparing sequencing results of the original tumor and the periesophageal lesion.

There were eight cases in which the sequencing panel was used to assess for PMD (Fig 4B). In five cases, the physician indicated that the reports confirmed relapse/progression of AML, including one case of bone marrow–negative, extramedullary relapse in the esophagus (Fig 5D). There were three patients who were being assessed for PMD while in an apparent clinical remission. Two of the three patients did not show persistent AML via PMD assessment. The third patient showed three residual variants at low VAF (*CEBPA* [VAF = 4%], *SMC1A* [VAF = 4%], and *WT1* [VAF = 3%]), suggesting persistent disease.

### Patients With No Change in Treatment Regimens in Response to Sequencing Panels

There were 64 cases for which the physician noted no change in therapeutic plan on the basis of the report. For the majority of cases (n = 35), the physician did not state a reason. Of those stated reasons, the most common was that no actionable variants were observed (n = 14; Fig 4B). There were 14 physicians who were surveyed for this study (Data Supplement). The total number of eligible surveys completed by each physician and the total number of cases where the physician changed his or her plan is shown in Figure 4C.

The sequencing panel reports the VAF and thus can detect possible inherited variants. One patient was found to have a *GATA2* variant observed at a VAF of 52%, indicative of a possible germline variant, which prompted an action by the treating physician. A subsequent skin biopsy determined that the patient did not have a germline predisposition for disease.

### Multiple Sequencing Panels for Longitudinal Disease Monitoring and Response

There were 12 patients for whom the treating physician ordered multiple reporters during the disease course^21^ (Fig 5 and Data Supplement). Figure 5A shows an example of how persistent variants resulted in a therapeutic influence. After induction, despite some variants demonstrating response to initial therapy, the *IDH2* VAF of 39% (46% at diagnosis) resulted in initiation of enasidenib. Figure 5B shows a patient for whom the physician obtained three sequencing panels for longitudinal PMD monitoring. The first report was ordered at relapse post-transplant and showed five variants. Two months later, a subsequent report showed no PMD. A month later, the third report showed the re-emergence of three variants despite no excess blasts on the bone marrow biopsy, which resulted in a second alloHCT. Figure 5C shows how a physician used sequential reports to observe the patient’s molecular response to a donor lymphocyte infusion and gilteritinib. Figure 5D shows how the reports diagnosed extramedullary relapse in a patient with negative bone marrow biopsy findings. The extramedullary disease showed the presence of some original variants (*NF1* and *EZH2*), loss of other variants (*DNMT3A*), and three novel variants (*CUX1*, *PTPN11*, and *WT1*), some of which had clinical implications.^34,35^ Additional cases with multiple reports are provided in the Data Supplement.

### Reimbursement and Clinical Care Access

One of the biggest challenges for the widespread clinical implementation of NGS-based diagnostics has been inconsistent reimbursement from payers. Coverage and reimbursement for MyeloSeq was divided into inpatient testing (26%) and outpatient testing (74%), and 90% of outpatient testing was reimbursed across all payers. There was no specific pattern in terms of the diagnosis of rejected claims (Data Supplement).

## DISCUSSION

This study reports our institution’s experience using a targeted capture sequencing panel to inform clinical care in patients with AML. The sequencing panel was ordered most commonly at diagnosis, and the majority of influences reported by physicians (68%) were related to therapeutic interventions. Predictive influences expanded beyond the detection of variants using FDA companion diagnostics, such as prescribing hypomethylating agents for *TP53*^20^ or *TET2*^28,29^ variants. We observed that physicians often made decisions on the basis of multiple co-occurring variants at diagnosis, during disease assessment, and/or at multiple timepoints during the disease course.

This study suggests that physicians are increasingly relying on the evaluation of multiple co-occurring variants in parallel; therefore, simultaneous analysis of these genes is necessary. Furthermore, PMD monitoring after cytotoxic induction therapy and transplantation^21,36^ has prognostic value; therefore, the VAFs reported on the gene panel studied here were being used to supplement existing standard-of-care mechanisms for residual disease assessment. Although it is clear that physicians are using this test to measure and assess PMD, this test was not designed for measurable residual disease testing, and the limit of detection is not within the European LeukemiaNet guidelines for measurable residual disease assessment.^15,37^ The prognostic information provided by the detection of persistent variants, the optimal depth of clearance, and the required limit of detection are all areas of active investigation requiring additional study in prospective clinical trials.

There were several limitations associated with this study. Reports were evaluated over an 8-month period at a single academic institution among physicians with experience interpreting genomic results. In addition, not all surveys were returned contemporaneously and tests were ordered at different timepoints during the disease course (Fig 3). Whereas the proportion of cases with influences was not significantly different among timepoints (Data Supplement), influence types might be different between timepoints and should be evaluated further to understand the impact of disease timepoint on treatment decisions. Finally, each physician had a varied number of patients (range, one to 18 patients) with a large range in the number of cases reported to be influential (0% to 90%). Larger longitudinal, multi-institutional studies are needed to better understand interphysician variability in incorporating genomic data into clinical decision making.

This study evaluated reports from the first 8 months after launch of the diagnostic. Even though the diagnostic was relatively new at the studied institution, physician experience with ordering internal and external genomic LDTs was high. The physicians were also experienced in interpreting genomic data, having participated in large genomic trials,^20,21,38-41^ with many of them being clinical investigators themselves. Given that the influence of genomic data varies on the basis of physicians’ experience with genomic data,^6,7^ the results presented here might not extrapolate to institutions that are unfamiliar with ordering and interpreting sequencing-based diagnostics.

The sequencing panel has several additional limitations. The use of a tumor-only panel—that is, lack of a germline or normal reference sequence—prevented variants from being definitively called somatic. In addition, some highly relevant variants—for example, *CEBPA*—had a nonzero failure rate which could influence results. In future studies, it will be important to ascertain from physicians if any aspect of the test or the molecular report is unclear, biased, or difficult to interpret.

There are several outstanding questions in the field that could be addressed using a test similar to the one described in this paper. The prognostic value of residual *DNMT3A*, *TET2*, and *ASXL1* mutations after treatment is incompletely understood,^21,36,42^ and the difference between a residual leukemic clone versus rising clonal hematopoiesis^43^ needs additional study. In addition, longitudinal assessment of VAFs during treatment could predict response to and efficacy of targeted therapies. Our group is currently conducting a prospective clinical trial (ClinicalTrials.gov identifier: NCT02178241) to address some of these outstanding questions.

In summary, the results from this study suggest that variants and associated VAFs are being integrated into clinical care to influence therapy choices, stratify relapse risk, and measure persistent disease. These uses should be further evaluated in prospective trials to determine if decisions that are supplemented by genomic data can improve outcomes.

## Appendix

### Supplementary Methods: Panel Design and Processing

The panel is generally ordered for one of the following conditions: acute myeloid leukemia (AML), myelodysplastic syndrome (MDS), cytopenia, clonal cytopenia of undetermined significance, clonal hematopoiesis of indeterminate significance, myeloproliferative neoplasm, or other myeloid disorders. Specimens were obtained from bone marrow aspirates, peripheral blood, or DNA extracted from fresh tissue. All steps of sample processing were performed in a clinical diagnostic laboratory that is College of American Pathologists accredited and Clinical Laboratory Improvement Amendments licensed. Clinical diagnosis, including disease status, was defined by board-certified hematopathologists.

The panel uses an amplicon capture-based enrichment with unique molecular identifiers for ultra-high variant sensitivity that targets an approximately 98-kg base pair space (HaloPlex Target Enrichment System; Agilent Technologies, Santa Clara, CA). The panel is generally ordered for one of the following conditions: AML, MDS, cytopenia, clonal cytopenia of undetermined significance, clonal hematopoiesis of indeterminate significance, myeloproliferative neoplasm, or other myeloid disorders. Specimens were obtained from bone marrow aspirates, peripheral blood, or DNA extracted from fresh tissue. All steps of sample processing were performed in a clinical diagnostic laboratory that is College of American Pathologists accredited and Clinical Laboratory Improvement Amendments licensed. Clinical diagnosis, including disease status, was defined by board-certified hematopathologists. Patients with a definitive diagnosis of AML were verified by evaluating the patient’s standard diagnostic workup. This included evaluating bone marrow morphology, standard panels of flow cytometric markers, and a panel of routine cytogenetic and molecular marker studies, including fluorescence in situ hybridization probes.

Target enrichment for the panel used a commercially available, targeted, next-generation sequencing approach (HaloplexHS; Agilent Technologies). Preparation consisted of enzymatic fragmentation; strand-specific ligation of sequencing primers, sample indexes, 10-bp degenerate molecular barcodes, and biotin tagging of single DNA molecules; rapid liquid-phase enrichment of target loci using paramagnetic streptavidin-coated beads; and on-bead polymerase chain reaction amplification. Sequencing was performed using the Illumina MiniSeq sequencing platform (target depth = 50× coverage).

Variant calling for the panel was performed using a computational pipeline that employs custom-built tools created at Washington University. Sequencer-generated FASTQ files were first demultiplexed and aligned to the reference genome (GRCh37). Overall reads were required to exceed 1,000,000 total reads with more than 98% aligned. During this process, unique molecular identifier sequences are added to the binary alignment map file. Once aligned, a custom Java-based tool collapsed reads into read families using customized GATK software (McKenna A, et al: Genome Res 20:1297-1303, 2010). The minimum read family size was five reads and consensus bases had to be in at least two thirds of the reads in the read family. Collapsed binary alignment maps were used with standard variant calling tools, including Varscan2 (Koboldt DC, et al: Genome Res 22:568-576, 2012), Platypus (Rimmer A, et al: Nat Genet 46:912-918, 2014), and Pindel (Ye K, et al: Bioinformatics 25:2865-2871, 2009). Quality control measures included a minimum mean unique coverage of 500 reads and more than 90% of positions within a targeted gene of more than 50× coverage. These programs detected single-nucleotide substitutions, insertions or deletions (indels) up to 10 bp, and *FLT3* internal tandem duplication insertions between 21 and 108 bp. Initial variant annotation was performed using the Variant Effect Predictor tool (McLaren W, et al: Genome Biol 17(1):122, 2016). Annotation was augmented using a custom variant call format file with variants identified in the AML The Cancer Genome Atlas data set as well as variants observed in three published sequencing reports that evaluated patients with MDS (Haferlach T, et al: Leukemia 28:241-247, 2014; Papaemmanuil E, et al: Blood 122:3616-3627, quiz 3699, 2013; Walter MJ, et al: Leukemia 27:1275-1282, 2013). The final output from the Variant Effect Predictor annotation was an annotated variant call format file and a text file with variant information. Variant identification was subject to the following thresholds and cutoffs for reporting purposes: variants must be nonsynonymous, variants must have 2% minimum variant allele frequency and five variant reads with support on each strand (*FLT3* internal tandem duplication alleles require one read on each strand), amplicons must have at least five reads assigned to it during consensus binary alignment map formation, and potential somatic variants must have 0.1% maximum population allele frequency (MAX_AF across all populations; 1000 genomes [v.phase3; 1000 Genomes Project Consortium, et al: Nature 526:68-74, 2015] and gnomAD database [v.170228; Karczewski KJ, et al: Nature 581:434-443, 2020]) or presence in a custom MDS/AML variant database.

Tier 1 (variants with strong clinical significance) and tier 2 variants (variants with potential clinical significance), four filtered variants (ie, variant allele frequency < 2%), and variants of unknown significance were manually reviewed for potential clinical relevance. These variants were used to generate a clinical report that summarized all relevant findings. Information on the reports included interpretations from National Comprehensive Cancer Network guidelines, WHO recommendations, and outcome data from published, peer-reviewed studies that pertained to disease prognosis and therapeutic sensitivity/response.

## References

[1] Ray T Survey of precision oncology programs finds agreement on testing, divergence in care delivery.

[2] Stone RM Mandrekar SJ Sanford BL et al Midostaurin plus chemotherapy for acute myeloid leukemia with a FLT3 mutation N Engl J Med 377 454–464 2017 28644114 10.1056/NEJMoa1614359 PMC5754190

[3] Kim AS Bartley AN Bridge JA et al Comparison of laboratory-developed tests and FDA-approved assays for BRAF, EGFR, and KRAS testing JAMA Oncol 4 838–841 2018 29242895 10.1001/jamaoncol.2017.4021 PMC6145687

[4] Li MM Datto M Duncavage EJ et al Standards and guidelines for the interpretation and reporting of sequence variants in cancer: A joint consensus recommendation of the Association for Molecular Pathology, American Society of Clinical Oncology, and College of American Pathologists J Mol Diagn 19 4–23 2017 27993330 10.1016/j.jmoldx.2016.10.002 PMC5707196

[5] Starlinger J, Pallarz S, Ševa J (2018). Variant information systems for precision oncology. BMC Med Inform Decis Mak.

[6] Chow-White P, Ha D, Laskin J (2017). Knowledge, attitudes, and values among physicians working with clinical genomics: A survey of medical oncologists. Hum Resour Health.

[7] Weipert CM Ryan KA Everett JN et al Physician experiences and understanding of genomic sequencing in oncology J Genet Couns 27 187–196 2018 28840409 10.1007/s10897-017-0134-3 PMC5810555

[8] Gornick MC Ryan KA Scherer AM et al Interpretations of the term ‘actionable’ when discussing genetic test results: What you mean is not what I heard J Genet Couns 28 334–342 2019 30964581 10.1007/s10897-018-0289-6 PMC10558004

[9] Statz CM Patterson SE Mockus SM Barriers preventing the adoption of comprehensive cancer genomic profiling in the clinic Expert Rev Mol Diagn 17 549–555 2017 28402162 10.1080/14737159.2017.1319280

[10] Spencer DH Ley TJ Sequencing of tumor DNA to guide cancer risk assessment and therapy JAMA 319 1497–1498 2018 29634818 10.1001/jama.2018.2281

[11] Au CH, Wa A, Ho DN (2016). Clinical evaluation of panel testing by next-generation sequencing (NGS) for gene mutations in myeloid neoplasms. Diagn Pathol.

[12] Kurzrock R Colevas AD Olszanski A et al NCCN Oncology Research Program’s Investigator Steering Committee and NCCN Best Practices Committee Molecular Profiling surveys J Natl Compr Canc Netw 13 1337–1346 2015 26553764 10.6004/jnccn.2015.0163

[13] Cancer Genome Atlas Research Network Ley TJ Miller C et al Genomic and epigenomic landscapes of adult de novo acute myeloid leukemia N Engl J Med 368 2059–2074 2013 23634996 10.1056/NEJMoa1301689 PMC3767041

[14] Papaemmanuil E Gerstung M Bullinger L et al Genomic classification and prognosis in acute myeloid leukemia N Engl J Med 374 2209–2221 2016 27276561 10.1056/NEJMoa1516192 PMC4979995

[15] Döhner H Estey E Grimwade D et al Diagnosis and management of AML in adults: 2017 ELN recommendations from an international expert panel Blood 129 424–447 2017 27895058 10.1182/blood-2016-08-733196 PMC5291965

[16] O’Donnell MR Tallman MS Abboud CN et al Acute myeloid leukemia, version 3.2017, NCCN Clinical Practice Guidelines in Oncology J Natl Compr Canc Netw 15 926–957 2017 28687581 10.6004/jnccn.2017.0116

[17] Wu M, Li C, Zhu X (2018). FLT3 inhibitors in acute myeloid leukemia. J Hematol Oncol.

[18] Abou Dalle I DiNardo CD The role of enasidenib in the treatment of mutant IDH2 acute myeloid leukemia Ther Adv Hematol 9 163–173 2018 30013764 10.1177/2040620718777467 PMC6041864

[19] Nassereddine S Lap CJ Haroun F et al The role of mutant IDH1 and IDH2 inhibitors in the treatment of acute myeloid leukemia Ann Hematol 96 1983–1991 2017 29090344 10.1007/s00277-017-3161-0

[20] Welch JS Petti AA Miller CA et al TP53 and decitabine in acute myeloid leukemia and myelodysplastic syndromes N Engl J Med 375 2023–2036 2016 27959731 10.1056/NEJMoa1605949 PMC5217532

[21] Klco JM Miller CA Griffith M et al Association between mutation clearance after induction therapy and outcomes in acute myeloid leukemia JAMA 314 811–822 2015 26305651 10.1001/jama.2015.9643 PMC4621257

[22] Marcucci G Mrózek K Ruppert AS et al Abnormal cytogenetics at date of morphologic complete remission predicts short overall and disease-free survival, and higher relapse rate in adult acute myeloid leukemia: Results from Cancer and Leukemia Group B study 8461 J Clin Oncol 22 2410–2418 2004 15197203 10.1200/JCO.2004.03.023

[23] Chen Y Cortes J Estrov Z et al Persistence of cytogenetic abnormalities at complete remission after induction in patients with acute myeloid leukemia: Prognostic significance and the potential role of allogeneic stem-cell transplantation J Clin Oncol 29 2507–2513 2011 21555694 10.1200/JCO.2010.34.2873 PMC4874214

[24] Ivey A Hills RK Simpson MA et al Assessment of minimal residual disease in standard-risk AML N Engl J Med 374 422–433 2016 26789727 10.1056/NEJMoa1507471

[25] Washington University in St Louis MyeloSeq.

[26] O’Donnell MR Tallman MS Abboud CN et al Acute myeloid leukemia, version 2.2013 J Natl Compr Canc Netw 11 1047–1055 2013 24029121 10.6004/jnccn.2013.0127 PMC4161234

[27] Skidmore ZL Wagner AH Lesurf R et al GenVisR: Genomic visualizations in R Bioinformatics 32 3012–3014 2016 27288499 10.1093/bioinformatics/btw325 PMC5039916

[28] Bejar R Lord A Stevenson K et al TET2 mutations predict response to hypomethylating agents in myelodysplastic syndrome patients Blood 124 2705–2712 2014 25224413 10.1182/blood-2014-06-582809 PMC4208285

[29] Feng Y, Li X, Cassady K (2019). TET2 function in hematopoietic malignancies, immune regulation, and DNA repair. Front Oncol.

[30] Perl AE Availability of FLT3 inhibitors: How do we use them? Blood 134 741–745 2019 31243041 10.1182/blood.2019876821

[31] Stone RM Manley PW Larson RA et al Midostaurin: Its odyssey from discovery to approval for treating acute myeloid leukemia and advanced systemic mastocytosis Blood Adv 2 444–453 2018 [Erratum: Blood Adv 2:787, 2018] 29487059 10.1182/bloodadvances.2017011080 PMC5858474

[32] Stein EM DiNardo CD Pollyea DA et al Enasidenib in mutant *IDH2* relapsed or refractory acute myeloid leukemia Blood 130 722–731 2017 28588020 10.1182/blood-2017-04-779405 PMC5572791

[33] Loken MR Alonzo TA Pardo L et al Residual disease detected by multidimensional flow cytometry signifies high relapse risk in patients with de novo acute myeloid leukemia: A report from Children’s Oncology Group Blood 120 1581–1588 2012 22649108 10.1182/blood-2012-02-408336 PMC3429302

[34] Hou H-A Chou WC Lin LI et al Characterization of acute myeloid leukemia with PTPN11 mutation: The mutation is closely associated with NPM1 mutation but inversely related to FLT3/ITD Leukemia 22 1075–1078 2008 17972951 10.1038/sj.leu.2405005

[35] Gaidzik VI Schlenk RF Moschny S et al Prognostic impact of WT1 mutations in cytogenetically normal acute myeloid leukemia: A study of the German-Austrian AML Study Group Blood 113 4505–4511 2009 19221039 10.1182/blood-2008-10-183392

[36] Jongen-Lavrencic M Grob T Hanekamp D et al Molecular minimal residual disease in acute myeloid leukemia N Engl J Med 378 1189–1199 2018 29601269 10.1056/NEJMoa1716863

[37] Schuurhuis GJ Heuser M Freeman S et al Minimal/measurable residual disease in AML: A consensus document from the European LeukemiaNet MRD Working Party Blood 131 1275–1291 2018 29330221 10.1182/blood-2017-09-801498 PMC5865231

[38] Mardis ER Ding L Dooling DJ et al Recurring mutations found by sequencing an acute myeloid leukemia genome N Engl J Med 361 1058–1066 2009 19657110 10.1056/NEJMoa0903840 PMC3201812

[39] Ley TJ Ding L Walter MJ et al DNMT3A mutations in acute myeloid leukemia N Engl J Med 363 2424–2433 2010 21067377 10.1056/NEJMoa1005143 PMC3201818

[40] Walter MJ Shen D Ding L et al Clonal architecture of secondary acute myeloid leukemia N Engl J Med 366 1090–1098 2012 22417201 10.1056/NEJMoa1106968 PMC3320218

[41] Ding L Ley TJ Larson DE et al Clonal evolution in relapsed acute myeloid leukaemia revealed by whole-genome sequencing Nature 481 506–510 2012 22237025 10.1038/nature10738 PMC3267864

[42] Spencer DH Russler-Germain DA Ketkar S et al CpG island hypermethylation mediated by DNMT3A is a consequence of AML progression Cell 168 801–816.e13 2017 28215704 10.1016/j.cell.2017.01.021 PMC5328582

[43] Xia J Miller CA Baty J et al Somatic mutations and clonal hematopoiesis in congenital neutropenia Blood 131 408–416 2018 29092827 10.1182/blood-2017-08-801985 PMC5790127

[44] McLaren W, Gil L, Hunt SE (2016). The Ensembl Variant Effect Predictor. Genome Biol.

